# Observations on Select Subjects in Surgery

**Published:** 1805-12

**Authors:** 


Medical and Physical Journal.

VOL. XIV.] DECEMBER, 1805. [NO. 82.*

*Printed for R. PHILLIPS, by W. Thorne, Red Lion Court, Fleet Street, London.*

### Observations on select Subjects in Surgery. By Mr. Simmons.

( Continued from Vol. xiii. pp. 385—390. )

### On Gutta Serena.

JAMES HEYWOOD, æt. 33, by trade a cotton-spinner, was admitted into the Manchester Infirmary on July 15, 1805, for a gutta serena of the right eye, which had rendered him totally blind, he having lost the sight of the left by the small-pox thirty years before.

Impaired vision, and a slight pain on motion at the bottom of the orbit, first indicated the approaches of this complaint, which came on one Saturday, and on the Tuesday following he was quite dark. When admitted, his appetite was good, and strength unreduced; his head was free from pain, and the pain in the eye had ceased for several days; but the pupil was dilated, and insensible to the strongest light.

I directed electric sparks to be drawn from the eye every day, as strong as he could well bear them; and at my next visit on the third, the pupil had regained some portion of mobility. Continuing the electrification, he was, in addition to it, now advised to take a dose of bark twice a day, and to go into the cold bath two or three times a week. His amendment was still progressive, near objects appearing to him better defined, and distant ones coming gradually within his range. To give still further aid to these powerful means, he next took a quarter of a grain of the hydrargyrus vitriolatus mixed up with a little liquorice powder for a sternutatory; and, although the irritation produced by it was considerable, and the discharge from the nose copious for about an hour after each taking, as he bore the operation well, the quantity was ordered to be repeated morning and evening. In this course he persevered with increasing advantage until he was dismissed

 cured cured on the 26th of August, after a stay in the house of five weeks.

The treatment in this case was founded upon an opinion of a palsy of the retina, or expansion of the optic nerve, not originating in compression, either from a tumour within the brain, or any local congestion. In such a case electricity has of late years been usually resorted to as a remedy; and here its efficacy was early conspicuous. To it the bark and cold bathing were subsequently joined as auxiliaries; and afterwards the mercurial snuff recommended in the gutta serena by Mr. Ware, in order to accelerate their good effects in a still quicker progression.

The late Dr. Heberden, in his excellent Commentaries, p. 281, speaks coldly of the utility of electricity in this disease; his words are, 'Aiunt electrificationem interdum fuisse utilem;' but perhaps his own trials of it had not been numerous; or cases in their nature irremediable had fallen under his observation. In the design of augmenting tone, the bark and cold-bath held an appropriate rank; and the mercurial snuff had a sufficient authority in the recommendation of Mr. Ware. In the opinion of Dr. Hope *senior*, the hydrargyrus vitriolatus, 'in doses of one grain, with a little powder of liquorice root, is the most convenient errhine he has had occasion to employ.'—Edinburgh New Dispensatory, p. 471, 1803. But experience has taught me, that one-fourth of a grain, diluted as above, operates in a very powerful manner.

From the history of this case it would appear, that evacuation of any kind was not indicated; or, as the loss of vision was sudden, and a short interval only had elapsed before he applied for assistance, I should have premised at least a single dose of a cathartic, agreeably to the advice of Celsus, the passage relating to which I shall here transcribe:—'Quidam sine ulla manifesta causa subito obcæcati sunt. Ex quibus nonnulli cum aliquandiu nihil vidissent, repentina profusione alvi lumen receperunt. Quo minus alienum videtur, et recenti re, et interposito tempore, medicamentis quoque moliri dejectiones, quæ omnem noxiam materiam in inferiora depellant.' (Lib. VI. chap. vi. p. 368, L. B.)

Under the term *mydriasis*, the older writers have comprehended a morbid affection of the iris, and of the retina. The appellation itself is expressive of humidity, on an increase of which in the eye, the pupil still continuing transparent, they supposed the cause of blindness in this instance to depend. A better acquaintance with the anatomical mical structure of this organ has cleared up this uncertainty, and proved that the iris may be affected while the retina is sound; and that a disease of the retina may exist independantly of any morbid affection of the iris. If the iris should have become immovable, and not entirely closed, in a sound state of the retina, vision will still be distinct at a given focal distance; but if the retina should have lost its sensibility, as through it the iris is secondarily acted upon, the pupil will dilate, and continue dilated, though not morbidly affected, for want of the necessary excitement.

When gutta serena is the symptom only of another complaint, the cure of the original malady must precede any attempt to restore the impaired functions of the nerves. For, notwithstanding the removal of the offending cause, insensibility, or torpor, is too often the consequence of any violence done to the nervous system.

In several cases of gutta serena I have experienced the beneficial effects of the vitriol of copper; the dose has been from a quarter to the third of a grain twice a day, mixed up with a little aromatic powder. In a week or ten days it has restored sight to several, without any sensible operation. But the subjects of these experiments were young, boys from nine to twelve years of age, and in each the disease had been of short duration. It may be proper likewise to observe, that I had no reason to suppose that any of these were instances of the mydriasis *verminosa*; and perhaps any other tonic might have wrought a cure.

### Impervious Funis Umbilicalis.

Whatsoever diversity of sentiment may have obtained among medical writers relative to the uterine state of the fœtus, all are agreed that the funis umbilicalis is the medium of communication between it and the mother. Thro' this medium the circulation of the blood, an office essentially necessary to the fœtal existence, is maintained between them; and any direct agency upon the fœtus, thro' the sensorium of its parent, is wisely guarded against by the absence of nerves in the structure of the funis.

Among the causes of abortion, or premature delivery, preceded by the death of the fœtus, the obliteration of this channel may therefore be justly enumerated, and it is probably a more frequent cause of that event than has been hitherto imagined. This opinion is founded on the case of a lady, whose long and severe sufferings in differ-

 ent ent pregnancies, had excited more than ordinary attention and concern. She had borne three living children, the birth of each of which was denoted by great irritability of habit. In the seventh month of her fourth pregnancy she was seized with convulsions, and early on the morning of the second day, as the os uteri had dilated, the membranes were ruptured, soon after which a dead child was expelled by the pains, loose and putrescent in its texture, and not larger than is commonly observed between the fourth and fifth months. At this birth the secundines were not particularly examined.

About the sixth month of her next pregnancy, she had another still-birth; this was preceded by threatening symptoms of convulsions, and the child, of which she had never felt the motion, apparently had not grown after the end of the fourth month. The after-birth was now examined, to see if any thing presented in it sufficient to account for the early death of the fœtus, and its premature delivery, when it was discovered that two inches from the body of the child the funis umbilicalis was impervious, and had assumed a ligamentous appearance. At the second, the third, and the fourth births following, each of which was still, and occurred at the distance of from one to two years from the other, a similar morbid alteration of structure in the funis was observed; the living principle had become extinct in the fœtus about the same period of pregnancy, and in each the expulsion of it had ensued at or soon after the expiration of the sixth month. In the third of the above pregnancies, symptoms of hemiplegia were threatening, but by timely assistance they were arrested in their progress. The fifth and last pregnancy (in the year 1803) she was a second time seized with puerperal convulsions at the usual period of gestation, which were again suppressed; and the fœtus, in a state verging to putridity, as in the former instance, was again expelled about the end of the sixth month. At every examination of the placenta it was found loose and collapsed; smooth on its adherent surface; and the separation of it had been so gradually effected, that the lochial discharge on its expulsion was hardly tinged sanguine. Each interval from the death of the fœtus to the birth of it, her feelings were extremely irritable; otherwise she was upon the whole tolerably well. The birth each time was natural, easy, and expeditious, and, except the first, quite free from any alarm of convulsions. In the last convulsive attack, it was thought expedient to rupture the membranes, and while she she lay insensible, I proceeded in the most cautious manner to rupture them; but the convulsive contortions were so greatly aggravated by the attempt, that I thought it prudent to desist.

In the medical treatment of this case our chief reliance was placed on opium, 130 drops of the tincture of which she took within an hour, during the first paroxysms of convulsions, and was kept under the gentle influence of it for several days, even after the convulsions had ceased. The muscles of the cheeks were rigidly contracted, and the jaws locked, so that the liquid was conveyed to the gullet through the space whence a tooth had been extracted, and she swallowed it without much difficulty. Other auxiliary remedies were employed, cold water was dashed on the face without abating the severity of the paroxysms, mustard poultices were applied to the feet, and blisters successively on the temples, between the shoulders, and to the calves of the legs. The threatening of hemiplegia was accompanied with symptoms of congestion in the head, and leeches were applied to the temples with manifest advantage. For the same reason, she was leeched again the last attack of convulsions, and also used the warm bath. Her health was undermined by these severe shocks on the nervous system, and her mental faculties weakened. A few months since she was seized with a spasmodic affection at the stomach, which, after a slight remission, was renewed with so much violence on the second day following, as to carry her off in a few seconds of time.

The body was not permitted to be opened after death.

An impervious funis umbilicalis is enumerated by writers on midwifery, among the causes of the death of the fœtus in utero; but an instance of the recurrence of it so often in the same subject has not, I believe, been before recorded. A knowledge of the function yielded by the placenta before birth being similar to that of the lungs after, will enable us to account satisfactorily for the death of the fœtus from this cause, for the closure of the funis must deprive it of the benefits of respiration, as effectually as a cord twisted tightly round the neck after it had been brought into the world.

It has been said that 'women are far more liable to convulsions in a first than in subsequent labours,' a position not confirmed by the case of this lady, who had previously born three living children. Nor could the death of the fœtus in any be ascribed to the convulsions; and in

 this this respect too the present case comes as an exception. 'When women have convulsions,' says Dr. Denman, 'the death of the children ought generally to be esteemed rather an effect than a cause.'* Here the accession of the convulsions, as well as it could be ascertained, was synchronous, or nearly so with the death of the fœtus, and demonstrably not the cause of its death.

But a much more important question is, whether in puerperal convulsions the patient should or should not be delivered by art; and as far as this lady's case can warrant an opinion, it is answered in the negative. This observation however, will not be meant to apply to that case of convulsions where the os uteri has dilated, and the only assistance required of art is to rupture the membranes. When I attempted to deliver during the closed state of the womb, the convulsions were alarmingly aggravated; hence we may conclude with Dr. Denman, that the treatment of the convulsions should be conducted without any reference to the labour, until the os uteri shall have dilated. At what period soever this event may happen, even should it be delayed, as in the present case, for many weeks after the death of the fœtus, then and not till then will be the proper time to give manual assistance.

The imperviousness of the funis umbilicalis is plainly beyond the reach of art. A similar contraction situated in an accessible tube, gifted with nervous power, could only be counteracted, even in an early stage of it, by mechanical distension. All then that is left us to do, is to improve and support the general health by suitable means; and, when our exertions fail of success, it is a consolation to know, that a remedy for the evil lay not within the scope of human ability.

### On Concussion, or Contusion of the Brain.

### Case I.

In April 1805, a stout man, twenty-nine years of age, was struck upon the left side of the chin by a thick broad deal plank, many yards in length, while assisting with others in lifting it through the window in the new building erecting in Charlotte Street. The plank rested at the time near its centre upon the sill of the window; the joists not being laid, the inner extremity descended suddenly into the cellar, and the outer one, which gave the blow, was carried up with the force and velocity of a lever. He fell speechless

* Introduction to Midwifery, quarto, p. 527.

speechless upon the ground, and in that state was immediately carried into the infirmary. It happening at the hour of my usual attendance, I examined him without delay, and found that he had received a wound over the posterior extremity of the right parietal bone about two inches in length, but that the bone was not fractured, nor denuded of pericranium. His left side however was paralytic, and his right side violently convulsed; his breathing was stertorous, and pulse slower than natural, and oppressed; the pupils displayed scarcely any sensibility to light. Copious bleeding from the right jugular vein relieved his breathing, and rather abated the force of the convulsions. Means were next used to empty the intestines, which however were so torpid that no evacuation from them could be procured before the fourth day, notwithstanding strong purgatives were exhibited both by the mouth and by glyster. On the seventh from the accident he had another motion, after which he had no more. On the third day the pulse was as low as 58 beats in the minute, yet he had so far recovered his senses, as to give a tolerably distinct answer to a simple question put to him. From this time the pulse gradually increased in frequency until it had risen to upwards of 130 strokes in the minute, the convulsions on the right side, and paralysis on the left stil continuing. During the course of the disease he was bled both generally and topically, as far as the pulse and constitution would bear, and blisters were applied alternately to the head, the temples, between the shoulders, to the thighs, and sinapisms to the feet, that a continual irritation might be kept up upon the surface of the body. But all were ineffectual, and on the ninth day he expired.

After death the vessels which pass over the upper surface of the brain were found turgid, and the right ventricle was filled with a large coagulum of blood.

### Case II.

A middle aged man was knocked down by a blow upon the head by a large mill-gate. I saw him soon after, when he was insensible, his breathing stertorous, his pulse slow, feeble, and interrupted, his urine involuntary, and just before I saw him he had parted with an involuntary stool; his body also was universally cold; he had bled a little from the nose and ears, but the pupils were not dilated. Warmth was applied to the extremities, and ammonia in small quantities frequently exhibited, to restore the circulation, and renew the temperature. The head was likewise shaved, and carefully examined, without discovering any race of fracture or depression. As soon as the pulse would bear it, fourteen ounces of blood were taken from the arm, mustard poultices were then applied to the feet, and the whole head was covered over with a blistering plaster. The bowels were afterwards copiously emptied; and a dose of the tinct. anodyn. antimon. was exhibited every six hours. The pulse became less oppressed by this treatment, but, as the other symptoms continued, the temporal artery was opened, after which he seemed somewhat rational for a short time, though the pulse began to sink after he had lost about six ounces of blood. In this state of stupor he lay for several days longer, with a pulse as low as 63 at one examination, and then it became again accelerated, and his mind still less obscured; for he could answer properly to a question that might be replied to in a monosyllable. He was next seized with a pain in the left temple, and as the pulse was upwards of 100, a dozen leeches were applied to the temples, and the parts were then covered with a blister. Though a little hurried by this discipline, his complaints were sensibly relieved, and the following night he was able to retain his urine. On the third day after the accident, an incision was made down to the cranium, upon the spot where he flinched on a particular examination, by which a fissure was exposed at the lower extremity of the left parietal bone, and a separation of the lambdoidal suture leading into the additamentum; but the pericranium was not separated nor the bone depressed. On the tenth day, however, as he was in all respects worse, the head of a large trephine was applied so as to remove the whole of the fissured part. The dura mater was every where adherent, nor was there any extravasation between it and the cranium. But instead of the silvery hue, the dura mater was discoloured, and it was opened to remove any extravasation that might be underneath; none however was found, so that after securing an artery and a vein, which had been divided in making the incision, light dressings were applied over the whole. Henceforward the pulse fluctuated between 100 and 120, became more and more feeble, and hurried by slight external objects; a low delirium supervened, and on the twelfth day from receiving the injury he expired, without having experienced, at any period, either rigors, convulsions, or paralysis of the extremities. The pupils were all along contracted and sensible to the light. I have it in recollection that at one visit this man was so much better as to sit upright in bed, and take milk pottage with a spoon, spoon, on which day I cannot now mention, and the record of it is omitted in my notes.

After death the right hemisphere of the brain was found covered over with extravasated blood, partly fluid and partly grumous; a considerable quantity more also lay between the side of the hemisphere and the cranium, and under the middle and posterior lobes. On the left side a small extravasation was discovered under the anterior lobe of the brain, and a little again at each extremity of the falciform process. The separation of the suture had extended along the additamentum into the base of the skull, yet scarcely any thing that amounted to extravasation was observed in the course of it. The fissured portion was entirely removed by the operation.

I have placed this case under the head of *contusion*, because neither the fissure in the cranium, nor the separation of the suture, adequately account for the mischief that proved fatal. Derangement of function of the injured organ and extravasation, well known effects of contusion, were present in this case, and justify in it, and in the former, the change of appellation which I have proposed. The sensibility of the pupils in the second will not escape remark; nor the surprising change for the better, which at one time gave no unfavourable presage even of recovery. Considering the appearances exhibited in the brain, it is not a little extraordinary that death followed not instantaneously on the accident in both.

In the first case, the blow was given upon the left side of the chin, and the blood was effused into the right ventricle of the brain; the right side of the body was convulsed, and the left paralytic; the bowels were nearly deprived of their irritability, and the urine was insensibly discharged.

In the second case, the extravasation was also on the side opposite to where the blow was inflicted, though on the surface of the brain; there was less torpor of the intestines, the bladder kept its retentive faculty, and the pupils their power of motion. Does the irritating cause which produces convulsions act upon both ventricles, if the convulsions are universal? or upon either, if they are partial? Is the irritation confined to the ventricle on the side affected with convulsions, and the paralysis transferred to the opposite side?

Where the sensorium can bear the compression in the first instance, symptoms of increased action afterwards ensue, and plainly indicate the vigorous employment of antiphlogistic antiphlogistic means, which by lessening the inflammatory tension may prolong the existence of the patient after an accident, where the mischief has been too deep for any rational hope of recovery to be entertained.

In one case after a contusion of the brain, where the motion of the voluntary muscles was enfeebled, and the patient nearly deprived of the faculty of reminiscence, I instituted a trial of the comparative efficacy of electricity and galvanism, by drawing the sparks of each alternately, every day from the temples, and other parts of the head. He gave the preference to the galvanism, thinking himself more animated after it than after electrification, which therefore was discontinued. His recovery was slow; at length however his articulation became tolerably distinct, and he could walk without stumbling; previously he had required help in walking, yet he could run the length of a large ward without making a single false step, similar to one under intoxication, who, when he could not walk strait or without fear of falling, could run to some distance with swiftness and safety.

*Manchester, Oct.* 18, 1805.

